# Fluoride Levels in Saliva and Plaque following the Use of High Fluoride and Conventional Dentifrices- a Triple Blinded Randomised Parallel Group Trial

**DOI:** 10.1155/2019/1636209

**Published:** 2019-05-02

**Authors:** Sharon Vincent, Abi M. Thomas

**Affiliations:** Pedodontics and Preventive Dentistry, Christian Dental College, Ludhiana, Punjab 141008, India

## Abstract

**Context:**

The comparison of fluoride levels in saliva and plaque following the use of conventional, 2800 and 5000 ppm dentifrices for different time intervals up to 24 hours has not been explored.

**Aim:**

The aim of the present study was to assess salivary and plaque fluoride levels at different time intervals following the use of high fluoride dentifrices.

**Study Design:**

This randomised control trial was conducted on sixty adolescents between the age group of 16 and 18 years divided into three groups A, B, and C.

**Intervention:**

Subjects were asked to brush the occlusal surfaces of posterior teeth with one of the dentifrices for 2 min. Unstimulated whole saliva and plaque samples were collected at different time intervals. Fluoride levels were determined using SPADNS method. Data was collected and statistically analyzed using SPSS 20 Inc. by mixed repeated measure ANOVA.

**Results:**

A significant difference in fluoride levels was observed at different time intervals in both saliva (p=0.048) and plaque (p=0.03). The variance was low with time and concentration of the dentifrice used in saliva, whereas the magnitude of treatment was large for time (>0.25) but small for (<0.09) concentration in plaque.

**Conclusion:**

A significant difference in fluoride levels was observed at different time intervals in both saliva and plaque among the three groups. There was a positive correlation between fluoride levels in saliva and plaque. Both high fluoride dentifrices were effective in maintaining higher intraoral fluoride levels up to 12 hours and 5000 ppm up to 24 hours compared to conventional dentifrice.

## 1. Introduction

Since the discovery of its anticariogenic potential, fluorides have been at the forefront of preventive dentistry. This has led to a dramatic decline in the prevalence of dental caries globally. Among the available fluoride delivery systems, dentifrices are most commonly used [1]. The caries protection from fluoride (F) dentifrices is largely dependent on the efficiency of the fluoride delivery and its ability to increase intraoral fluoride levels [2, 3].

The higher the fluoride concentration available is, the greater its impetus to diffusion through the biofilm towards the tooth surface will be. Marihno et al. suggested that an increase of 500 ppm F (within the range of 1100–2500 ppm F) would amplify caries reduction potential by 6% [4]. Fluoride toothpastes with <1450 ppm F content have been reported to be less effective in high-risk children [5].

Dentifrices with higher concentrations of fluoride (2800 and 5000 ppm) have recently been introduced but are available only on prescription. Most of the clinical studies evaluating these toothpastes have focused on caries reduction. However, the comparison of fluoride levels in saliva and plaque has not been explored following use of dentifrices with high fluoride concentrations. The present study aims to assess plaque and salivary F levels at different time intervals up to 24 hours after the use of high fluoride dentifrice in varying concentrations in an optimally fluoridated community. The null hypothesis states that no difference exists in the saliva and plaque F concentration following dentifrice application.

## 2. Methods

### 2.1. Study Design

The triple blinded randomized parallel group trial has been reported in accordance with the CONSORT 2010 guidelines. The sample consisted of 60 adolescents between 16 and 18 years. The inclusion criteria required subjects with the following [6]:

1. A full complement of teeth (except for third molars and premolars extracted for orthodontic therapy)
2. DMFT score less than one
3. Mild or no gingivitis with an acceptable attachment loss of less than 2 mm
4. No evidence of a deeply fissured tongue or irregular oral mucosal surfaces which might enhance fluoride retention
5. Unstimulated salivary flow rates between 0.2 and 0.7 mL/min

The exclusion criteria excluded subjects [7]

1. with marked intraoral soft tissue pathology,
2. on an antibiotic regimen 3 months before or during the course of study,
3. with medically compromising conditions,
4. undergoing orthodontic therapy.

The sample size was calculated based on the findings from similar studies at 95% confidence interval. The desired statistical power was set at 80% (1 – *β* = 0.8) at a significance level of 5% (*α* < 0.05). Twenty subjects per group were required to avoid any statistical type I errors. Computer generated random numbers were used to allot subjects into three groups A, B, and C (1:1:1) depending on the concentration of fluoridated toothpaste used. The narrow age range helped minimize age related variations in salivary flow rates [6]. The final sample consisted of 60 subjects in the age group of 16-18 years, divided into three groups with a mean age of 16.9 ± 0.75 years. 65% of the study subjects were females and 35% were males. All participants were residents of an area with ≤1.2 ppm fluoride in drinking water. The concentration of the toothpaste used was masked from the principal investigator, the subjects and the statistician ensuring triple blinding.

### 2.2. Intervention

Two days prior the test, subjects received oral prophylaxis and polishing with a nonfluoridated prophylaxis paste (Protec, UK) for removal of hard and soft dental deposits and to eliminate any carried over effects from previous fluoride exposure. Subjects were instructed to use a non-fluoridated dentifrice and to avoid brushing facial surfaces of maxillary teeth and lingual surfaces of mandibular teeth for two days prior testing to permit plaque accumulation. Subjects were specially advised to refrain from using fluoride containing dental products and to abstain from eating foods and beverages high in fluoride, to prevent distortion of research results. Baseline saliva and plaque samples were collected. A 2 cm ribbon of Colgate® Total1000ppm fluoridated toothpaste or Colgate® Duraphat® 2800ppm fluoridated toothpaste or Colgate® Duraphat® 5000ppm fluoridated toothpaste was dispensed by a trained operator. Subjects were asked to brush only the occlusal surfaces in all four quadrants for 1min. Brushing of the facial and lingual tooth surfaces was avoided so that plaque could be collected subsequently. Immediately following brushing, the subjects were asked to rinse with 15 ml of tap water for 10 seconds [8].

#### 2.2.1. Saliva and Plaque Collection

Unstimulated salivary flow rates between 0.2 and 0.7 mL/min was the selected criterion for testing. The use of stimulated saliva was deterred as it would increase the rate of F clearance from the oral cavity, also artificially lowering the F levels in subsequent samples. During the first hour of the test, subjects were not permitted to eat or drink, and conversation was kept to the minimum. Unstimulated whole saliva samples were collected by passive drool method at baseline (0), 5, 15, 30, and 60 minutes and 2, 6, 12, and 24 hours after brushing.

The subjects were asked to swallow before plaque collection in each quadrant, to minimize saliva contamination. A Hollenbeck carver was used, with gentle scraping motion, for plaque sample collection from the facial surfaces of maxillary teeth and lingual surfaces of mandibular teeth. Plaque samples were weighed using a digital portable scale and 0.35-2.0 mg plaque samples were considered for the analysis. Higher and lower sample weights were omitted due to inconsistent test values. Immediately following collection, plaque samples were placed in plastic vials with 1 ml of de-ionized water [8]. Samples were labelled and transported to the laboratory on the same day for testing.

#### 2.2.2. Fluoride Analysis

The colorimetric SPADNS method was employed in the determination of fluoride concentrations, using a programmed spectrophotometer (Hach DR 2800). Sample to be tested was emptied into cuvettes (1-inch square, 10 mL) and deionized water was added to ensure a total volume of 10 ml.

Two milliliters of SPADNS reagents was added to these cuvettes. Determination of fluoride ion concentration was achieved at 570 nm wavelength by measuring the absorbance of an initial complex between zirconium ion and the dye, Trisodium 2-(4-sulfophenylazo)-1, 8-dihydroxynaphthalene-3, 6-disulfonate (SPADNS), and the subsequent bleaching of the complex, due to reaction with fluoride ion.

Interferences due to turbidity were not observed in this study due to relatively small quantities of saliva and plaque sampled with adequate dilution control. This test is usually sensitive to interferences from aluminum ions, which was confirmed by inconsistent readings with a few samples. Three consecutive readings were taken for such samples at 0 and 15 minutes and 2 hours, as per the manufacturer's instructions.

#### 2.2.3. Statistical Analysis

Mixed ANOVA for repeated measures followed by post hoc tests were used to compare the fluoride levels at different time intervals following the use of high fluoride dentifrices with different fluoride concentrations. Mauchly's sphericity test, along with Greenhouse-Geisser correction, was used to validate the results of mixed repeated measures (ANOVA). Logarithmic transformation of data was required as the fluoride levels at different time intervals were not following normal distribution according to Shapiro-Wilk test. Pair wise comparison was done by Bonferroni post hoc test (intragroup and intergroup) to identify the differences in fluoride levels between each pair of time intervals.

A statistical analysis software (SPSS 20 Inc. Chicago, IL, USA) was used and the results tabulated.

## 3. Results

**Figure 1:**
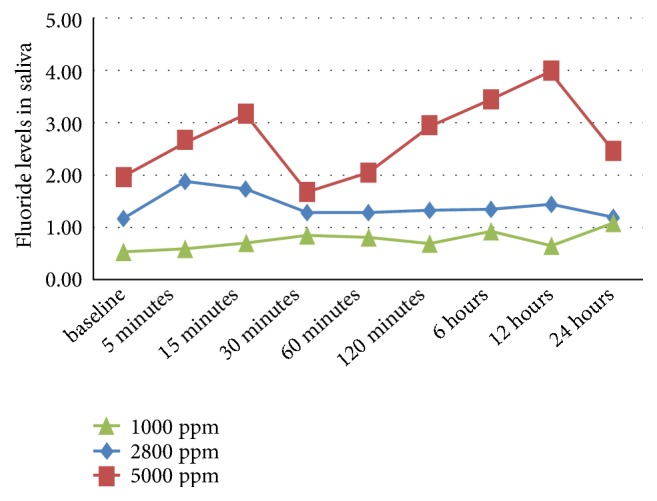
Variation in fluoride levels in saliva at different time points.

The fluoride levels in saliva at each time interval were compared among the three groups (Tables 1 and 2, Figure 1). A significant difference in fluoride levels was observed at different time intervals (p=0.048). Also, the change in fluoride levels was statistically significant in the three groups (P=0.008*∗*). The effect size, which measures the magnitude of treatment effect, was small for both time and group (<0.09). The variance was low with time and concentration of the dentifrice used.

An increase of 0.322 units of fluoride levels in saliva at 24 hours compared to the baseline was observed in all subjects irrespective of concentration. In Group C with 5000 ppm, an increase of 0.815 unit at 24 hours was observed, compared to 120 minutes. Both these results were statistically significant.

**Figure 2:**
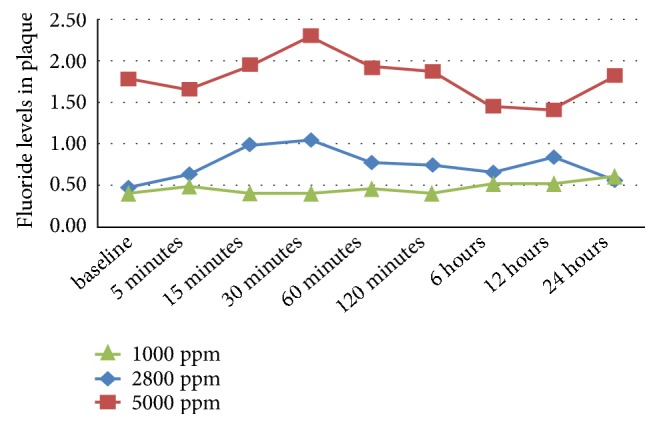
Variation in fluoride levels in plaque at different time intervals following the use of dentifrices with different fluoride concentrations.

Similarly, fluoride levels in plaque at each time-point were compared among the three groups by ANOVA (Tables 3 and 4, Figure 2). A significant difference was observed in fluoride levels in plaque at different time intervals (p=0.037). The change in fluoride levels in plaque was statistically significant between the three groups (P=0.03*∗*).

It was found that there was a statistically significant increase of fluoride levels in plaque at 24 hours (0.311 unit) compared to that in baseline in 60 subjects irrespective of concentration. Statistically significant values were observed on comparison between fluoride levels in saliva and plaque only in Group B at baseline, 5 min and 15 min (Table 5).

## 4. Discussion

The use of conventional fluoridated dentifrices (1000–1500 ppm F) has been a reliable therapeutic measure in reducing cariogenic potential. However, there exists a fraction of the population where the conventional F regimens minimally impact the caries rate. Davies and Davies (2008) stated that toothpastes containing higher fluoride concentrations exhibited an 18% reduction in incremental caries rates in comparison to toothpastes with 1,000 ppm F [9]. Studies have revealed that high fluoride toothpastes have a more preventive influence on caries in children, adolescents, and young adults when compared with a traditional toothpaste [4].

The findings of the present study indicate that the null hypothesis has been rejected and a positive dose-response relationship existed among the three groups of dentifrices in both saliva and plaque. There is a well-established dose-response relationship between the concentration of fluoride present in dentifrices and caries prevention and the evidence to support the same has been described by Cochrane as unequivocal [4].

In the present study an overall comparison showed a significant difference in fluoride levels at all-time intervals among all the groups. A Cochrane review analyzed fluoride toothpastes containing up to 2,800 ppm and described a dose-response effect up to this level but stated that statistically significant differences were not always observed between individual concentrations [10].

The fluoride levels in all groups followed a biphasic exponential pattern with an initial peak at 30 min in Group A (1000ppm), 5 min in Group B (2800ppm), and 15 min in Group C (5000ppm). The second peak was observed at 6 hours in 1000ppm group and at 12 hours in both 2800 and 5000 ppm groups. It is known that plaque and salivary F levels increase rapidly after the application of topical fluoride agents, following a biphasic, exponential pattern [11]. Naumova et al. observed a peak increase of salivary fluoride concentration immediately after brushing, which lasted for at least 30 minutes [12]. It was observed by Duckworth and Morgan that after brushing with a fluoride dentifrice, salivary fluoride decreased in two distinct phases: an initial phase lasting 40–80 min and a slow, second phase lasting several hours [3]. Hence, variable peaks and troughs were observed in the above-mentioned studies, probably due to the variations in the protocol followed.

The present study observed elevated salivary F levels on tooth brushing with high fluoride dentifrice, with effects lasting up to 12 hours after brushing. This phenomenon is due to the binding of fluoride to intraoral reservoirs and its subsequent release into saliva over time. Hence, it can be safe to say that 2800 and 5,000 ppm F toothpastes have a greater impact on individuals who do not use toothpaste regularly or do not brush twice a day.

Dentifrices containing sodium fluoride were used owing to its instant dissociation in saliva [12]. Subjects with unstimulated salivary flow rates between 0.2 and 0.7 mL/min were selected to minimize errors due to differential salivary flow rate. In order to standardize the fluoride exposure, tooth brushing with a two-centimeter ribbon of sodium fluoride dentifrice was done for 2 min followed by rinsing with 15 ml tap water [7]. The intraoral F retention or substantivity depends upon different factors such as salivary flow rate, age, stimulation effects, properties of fluoride containing products, volume and application time, vehicle of fluoride delivery, individual characteristics of saliva, and postbrushing rinsing behavior [6]. Hence, the above-mentioned factors were similar for all the participants in this study, though the differences in salivary kinetics remained specific to each participant.

Comparison of fluoride levels in saliva within each group from baseline showed statistically significant differences between fluoride levels at 6 hours and 12 hours in 1000 ppm group and at 5 mins in the 2800 ppm group. All other values were nonsignificant probably because of a higher baseline value in the 5000 ppm group despite the uniform protocol followed. High inter- and intraindividual variations in fluoride levels observed were similar to studies by Naumova et al. and Ekstrand et al. [12, 13].

A 2-fold rise in fluoride levels was observed on increasing F concentration 2.8 times (2800 ppm) and a 3.5-fold rise was observed with 5000 ppm in comparison to conventional dentifrices. Nordstrom et al. used fluoride concentrations of 1450 and 5000 ppm and reported an increase in bioavailability of fluoride by 37% [14].

In the present study, the concentration of F in the dentifrice greatly influenced F retention in plaque, conforming to published findings by Duckworth and associates [2]. The dose-response relationship, however, was nonlinear in the plaque group [3]. An overall comparison showed significant differences in fluoride levels in plaque at all-time intervals. A statistically significant difference was observed within Group A at 24 hours and at all-time points from 15 min up to 24 hours in Group B (2800ppm) compared to the baseline value. Fluoride bioavailability in plaque according to some studies from baseline increased after 30 min and was back to baseline after 6 hours [15]. On the contrary, few studies reported highly variable fluoride concentrations in plaque. In the present study, a rapid fluoride uptake was observed at 5 min in the 1000 ppm group, whereas, in Groups B and C, the fluoride levels increased after 30 min of brushing and were higher than the baseline values even after 12 hours. There is much evidence to establish a restricted fluoride-plaque uptake on short-term exposure (up to 120 seconds), whereas exposure for 30 min demonstrates significantly higher concentrations even in deep plaque layers towards the enamel surface [16]. The variability of literature on plaque fluoride is partly ascribed to analytical problems, many assays being close to or below the concentration detection limit. Measurements of fluoride in plaque are more difficult than in saliva because of the small amounts of sample collected.

The fluoride content of plaque, in this study, varied considerably between 0.2 ppm and 8 ppm. Appreciable variations in the fluoride content of saliva and plaque from similar fluoride areas have been observed by many investigators. Ionized fluoride in the plaque quickly diffuses to saliva or is adsorbed onto the enamel surface, thus being lost from plaque [17]. It must therefore be assumed that when these high fluoride concentrations are present, much of the fluoride must be in some form bound to the inorganic matter, such as calcium, or to organic matter. There are two recognized forms of fluoride retention in dental biofilms, both of which are related to calcium, i.e., precipitated minerals (calcium fluoride) and bacterially bound fluoride reservoirs. Application of high concentrations of fluoride (≥1000 ppm) leads to the formation of a calcium fluoride layer CaF_2_ on the enamel surface [18]. It has been reported that this CaF_2_ layer dissolves rapidly and releases bioavailable fluoride [19].

This clinical study demonstrated intraindividual and interindividual variability as well as differences in the salivary and plaque fluoride bioavailability similar to other studies by Naumova et al. [11, 12]. Statistically significant values were observed on comparison between fluoride levels in saliva and plaque in individual groups only in Group B at baseline, 5 mins, and 15 mins. All other values were nonsignificant. This might be due to the fact that plaque samples were collected from different sites at each time interval, in order to obtain a substantial amount of the sample. The discrepancy may also be due to the site-specific differences in fluoride levels in plaque, as well as differences in fluoride distribution between whole plaque, plaque fluid, and solid matter.

The plaque fluoride data was in qualitative agreement with corresponding salivary fluoride data implying that both are good indicators of changes in intraoral F levels when different F regimens are used. Vogel et al. and Duckworth et al. found that plaque fluid F and saliva F were correlated, irrespective of tooth collection site [3, 20] as observed in the present study. While initial fluoride concentrations were higher in saliva than those obtained in plaque, it is important to consider that this increase has low substantiality with time, due to continuous self-cleaning with saliva and other fluids. Plaque, acting as a fluoride reservoir, maintained a constant F level. Zero and associates established that mean whole plaque F clearance curves followed roughly similar profiles to corresponding saliva F curves up to 2 h after F application, though the larger variations observed between subjects for the plaque data [7].

## 5. Conclusion

High fluoride dentifrices could be considered effective in maintaining higher intraoral fluoride levels in comparison to conventional dentifrices. Therefore, the tooth brushing protocol using 1000 ppm may be considered for patients with low cariogenic risk and higher fluoride dentifrices (2800 ppm or 5000 ppm) may be prescribed for high risk patients. Following an initial caries risk assessment, 2800 ppm (age >10 years) and 5000 ppm (>16 years) may be recommended for optimally fluoridated and nonfluoridated communities, as a cost-effective method of caries prevention.

## Figures and Tables

**Table 1:** Intergroup comparison of fluoride levels in saliva at each time point.

TIME PERIOD	GROUP A	GROUP B	GROUP C	
	(1000 ppm)	(2800 ppm)	5000 ppm)	
	Mean ± SD	Mean ± SD	Mean ± SD	p value
0 min	*0.55* ± 0.54	*1.21* ± 1.54	*1.95* ±4.27	0.252
5 min	*0.60* ± 0.83	*1.8* ± 1.40	*2.66* ± 6.14	0.215
15 min	*0.71* ± 0.98	*1.67* ± 1.07	*3.2* ± 8.25	0.272
30 min	*0.87* ± 1.14	*1.23* ± 0.78	*1.68* ± 2.67	0.353
60 min	*0.83* ± 1.27	*1.27* ± 1.54	*2.05* ± 4.34	0.371
120min	*0.68* ± 1.23	*1.31* ± 2.04	*2.97* ± 7.49	0.268
6 hrs	*0.94* ± 1.03	*1.33* ± 1.79	*3.45* ± 8.80	0.272
12hrs	*0.67* ± 1.03	*1.35* ± 1.68	*4* ± 1.78	0.282
24hrs	*1.11* ± 1.40	*1.12* ± 1.50	*2.46* ± 5.45	0.372

**Table 2:** Comparison of fluoride levels in saliva within each group from baseline.

Time Period	Group-A		Group-B		Group-C	
	(1000ppm)		(2800ppm)		(5000ppm)	
	Mean ± SD	p-value	Mean ± SD	p-value	Mean ± SD	p-value
0 min	*0.55* ± 0.54	from 0min	*1.21* ± 1.54	from 0min	*1.95* ± 4.27	from 0min
5min	*0.60* ± 0.83	0.508	*1.8* ± 1.40	*0.022*	*2.66* ± 6.14	0.155
15min	*0.71* ± 0.98	0.170	*1.67* ± 1.07	0.061	*3.2* ± 8.25	0.187
30min	*0.87* ± 1.14	0.079	*1.23* ± 0.78	0.923	*1.68* ± 2.67	0.577
60min	*0.83* ± 1.27	0.137	*1.27* ± 1.54	0.780	*2.05* ± 4.34	0.684
120min	*0.68* ± 1.23	0.510	*1.31* ± 2.04	0.687	*2.97* ± 7.49	0.180
6Hrs	*0.94* ± 1.03	*0.010*	*1.33* ± 1.79	0.486	*3.45* ± 8.80	0.168
12hrs	*0.67* ± 1.03	0.334	*1.35* ± 1.68	0.348	*4* ± 1.78	0.242
24hrs	*1.11* ± 1.40	*0.020*	*1.12* ± 1.50	0.921	*2.46* ± 5.45	0.109

*∗*Statistically significant at *α*=5%.

**Table 3:** Intergroup comparison of fluoride levels in plaque at each time point.

TIME PERIOD	GROUP A	GROUP B	GROUP C	
	(1000 ppm)	(2800 ppm)	5000 ppm)	
	Mean ± SD	Mean ± SD	Mean ± SD	p value
0 min	*0.43* ± 0.59	*0.45* ± 0.29	*1.77* ± 2.82	*0.019*
5 min	*0.50* ± 0.80	*0.64* ± 0.54	*1.65* ± 3.38	0.155
15 min	*0.41* ± 0.60	*0.91* ± 0.54	*1.95* ± 4.74	0.211
30 min	*0.40* ± 0.71	*1.01* ± 0.54	*2.30* ± 6.10	0.237
60 min	*0.47* ± 0.68	*0.76* ± 0.41	*1.92* ± 4.36	0.175
120min	*0.41* ± 0.71	*0.74* ± 0.44	*1.87* ± 3.93	0.122
6 hrs	*0.54* ± 0.85	*0.66* ± 0.41	*1.46* ± 2.51	0.133
12hrs	*0.53* ± 0.74	*0.83* ± 0.59	*1.40* ± 2.06	0.111
24hrs	*0.60* ± 0.79	*0.54* ± 0.27	*1.82* ± 2.32	*0.009*

**Table 4:** Comparison of fluoride levels in plaque within each group from baseline.

Time Period	Group-A		Group-B		Group-C	
	Mean	p-value	Mean	p-value	Mean	p-value
0min	*0.43* ± 0.59	From baseline	*0.45* ± 0.29	from baseline	*1.77* ± 2.82	from baseline
5min	*0.50* ± 0.80	0.319	*0.64* ± 0.54	0.060	*1.65* ± 3.38	0.815
15min	*0.41* ± 0.60	0.821	*0.91* ± 0.54	*0.001*	*1.95* ± 4.74	0.810
30min	*0.40* ± 0.71	0.774	*1.01* ± 0.54	*0.001*	*2.30* ± 6.10	0.613
60min	*0.47* ± 0.68	0.445	*0.76* ± 0.41	*0.001*	*1.92* ± 4.36	0.827
120min	*0.41* ± 0.71	0.839	*0.74* ± 0.44	*0.004*	*1.87* ± 3.93	0.872
6Hrs	*0.54* ± 0.85	0.217	*0.66* ± 0.41	*0.010*	*1.46* ± 2.51	0.493
12hrs	*0.53* ± 0.74	0.251	*0.83* ± 0.59	*0.004*	*1.40* ± 2.06	0.410
24hrs	*0.60* ± 0.79	*0.004*	*0.54* ± 0.27	*0.041*	*1.82* ± 2.32	0.802

**Table 5:** Comparison between fluoride levels in saliva and plaque in Group B.

Time Period	Saliva		Plaque		
	Mean	SD	Mean	SD	p-value
0 min	1.21	1.54	0.45	0.29	*0.036*
5min	1.80	1.40	0.64	0.54	*0.001*
15min	1.67	1.07	0.91	0.54	*0.007*
30min	1.24	0.78	1.01	0.54	0.280
60min	1.25	1.54	0.76	0.41	0.177
120min	1.31	2.04	0.74	0.44	0.225
6Hrs	1.33	1.79	0.66	0.41	0.108
12hrs	1.36	1.68	0.83	0.59	0.192
24hrs	1.20	1.50	0.54	0.27	0.061

## Data Availability

The data used to support the findings of this study are available from the corresponding author upon request.
